# Linking Spatial Structure and Community-Level Biotic Interactions through Cooccurrence and Time Series Modeling of the Human Intestinal Microbiota

**DOI:** 10.1128/mSystems.00086-17

**Published:** 2017-09-05

**Authors:** Eric J. de Muinck, Knut E. A. Lundin, Pål Trosvik

**Affiliations:** a Centre for Ecological and Evolutionary Synthesis, Department of Biosciences, University of Oslo, Oslo, Norway; b Department of Gastroenterology, Oslo University Hospital–Rikshospitalet, Oslo, Norway; Dalhousie University

**Keywords:** 16S rRNA gene, microbiome, biotic interactions, intestine, microbial ecology, spatial structure, time series

## Abstract

The gastrointestinal (GI) microbiome is a densely populated ecosystem where dynamics are determined by interactions between microbial community members, as well as host factors. The spatial organization of this system is thought to be important in human health, yet this aspect of our resident microbiome is still poorly understood. In this study, we report significant spatial structure of the GI microbiota, and we identify general categories of spatial patterning in the distribution of microbial taxa along a healthy human GI tract. We further estimate the biotic interaction structure in the GI microbiota, both through time series and cooccurrence modeling of microbial community data derived from a large number of sequentially collected fecal samples. Comparison of these two approaches showed that species pairs involved in significant negative interactions had strong positive contemporaneous correlations and vice versa, while for species pairs without significant interactions, contemporaneous correlations were distributed around zero. We observed similar patterns when comparing these models to the spatial correlations between taxa identified in the adherent microbiota. This suggests that colocalization of microbial taxon pairs, and thus the spatial organization of the GI microbiota, is driven, at least in part, by direct or indirect biotic interactions. Thus, our study can provide a basis for an ecological interpretation of the biogeography of the human gut.

**IMPORTANCE** The human gut microbiome is the subject of intense study due to its importance in health and disease. The majority of these studies have been based on the analysis of feces. However, little is known about how the microbial composition in fecal samples relates to the spatial distribution of microbial taxa along the gastrointestinal tract. By characterizing the microbial content both in intestinal tissue samples and in fecal samples obtained daily, we provide a conceptual framework for how the spatial structure relates to biotic interactions on the community level. We further describe general categories of spatial distribution patterns and identify taxa conforming to these categories. To our knowledge, this is the first study combining spatial and temporal analyses of the human gut microbiome. This type of analysis can be used for identifying candidate probiotics and designing strategies for clinical intervention.

**Author Video**: An author video summary of this article is available.

## Abstract (precis)

The human gut microbiome is the subject of intense study due to its importance in health and disease. The majority of these studies have been based on the analysis of feces. However, little is known about how the microbial composition in fecal samples relates to the spatial distribution of microbial taxa along the gastrointestinal tract. By characterizing the microbial content both in intestinal tissue samples and in fecal samples obtained daily, we provide a conceptual framework for how the spatial structure relates to biotic interactions on the community level. We further describe general categories of spatial distribution patterns and identify taxa conforming to these categories. To our knowledge, this is the first study combining spatial and temporal analyses of the human gut microbiome. This type of analysis can be used for identifying candidate probiotics and designing strategies for clinical intervention.

## INTRODUCTION

As has been well documented, the gastrointestinal (GI) tracts of humans contain a complex ecology of microorganisms with intimate and often long-term interactions with the host (1). The many recent studies of the human GI microbiome have increased our knowledge of this relationship and its impacts on our development, physiology, immune system, and nutrition (2). However, much work remains to be done in order to come to an even partial understanding of this complex and dynamic ecosystem and its spatial distribution along the intestinal tract. The challenges in this area are due to several factors, including the high number of potentially interacting species, heterogeneity of habitats along the GI tract, and inherent difficulties of sampling (3). Additionally, and perhaps most importantly, there are large individual and temporal variations in microbiome composition (2).

Most studies rely on one or a few easily obtained fecal samples in order to profile an individual’s gut microbiome (3). Individual variation seems to be the most important factor in microbiome compositions, and this supports the use of fecal samples to characterize and compare individuals. However, there is much evidence that fecal sampling may not represent the adherent microbial communities (4). The GI tract has gradients of pH, oxygen availability, and host factors (2). Given this, there have been conflicting reports as to the biogeographical distribution of members of the GI microbiota, with some finding evidence to support spatial segregation (5, 6) while others did not see differences between the different anatomical regions (7, 8).

The personalized and dynamic ecosystem that comprises the human GI microbiome is influenced by the host environment and by the interspecies bacterial interactions themselves (9). Ecological interactions within these GI communities, while distinct and specific to the individual, display universality in that aspects of the interspecies interactions may be generalizable (10). These findings highlight the importance of studying the microbial interactions at the individual level and relating these to the GI biogeography.

In order to address some of these issues and investigate the relationship between microbial interactions in the human GI tract and how these interactions could relate to the spatial segregation of the bacterial taxa along the GI tract, we obtained 139 daily fecal samples from a single individual (main study individual), as well as triplicate biopsy samples from the same individual from seven sites along the GI tract at a single time point. To our knowledge, this is the first study linking spatial cooccurrence of microbial taxa along the GI tract with the biotic interactions estimated by time series analysis. By profiling the relative bacterial/archaeal abundances in these samples using 16S rRNA gene amplicon sequencing, followed by time series analysis as described in references 9 and 11, we were able to determine interacting taxa. From the biopsy sampling, we observed six different categories of spatial distributions along the GI tract. We then compared the taxa that were found to be significantly interacting from the time series analysis with the cooccurrence information from the biopsy samples and found that interacting species were significantly more likely to cooccur. These findings shed light on the ecological segregation along a gastrointestinal tract and the relationship between these communities and the more easily obtainable fecal samples. The duration and frequency of sampling allowed for relating cooccurrence modeling with time series analysis in that very similar information can be obtained, but for both approaches, it is necessary to have a relatively large number of samples. Both cooccurrence and time series modeling do provide very similar information about which taxa are involved in pairwise interactions; however, time series modeling can describe the asymmetry of these interactions much more fully. Overall, these findings can be used for future experimental designs that address both the complexities of biotic interactions in GI microbial communities and the spatial distribution of community members.

## RESULTS

### Overall diversity and spatial structure along the intestinal tract.

Biopsy samples were obtained, in triplicate, from a healthy adult at seven sites along the GI tract: terminal ileum (TI), ileocecal valve (IV), ascending colon (AC), transverse colon (TC), descending colon (DC), sigmoid colon (SC), and rectum. We further obtained 139 daily fecal samples from the same individual collected both before and after biopsy sampling. 16S rRNA gene amplicon sequencing of the 160 samples generated a total of 22,302,434 reads. The mean read number per sample was 139,390 (standard deviation [SD], 71,119). A total of 2,238 operational taxonomic units (OTUs) were found using the criterion of 97% sequence identity for clustering to an OTU (see Table S1 in the supplemental material). The mean read depth of the biopsy samples was lower (59,831 ± 39,922 [SD]) than for the fecal samples. We attribute the lower number of reads to the relatively small amount of sample material in the biopsy samples.

10.1128/mSystems.00086-17.9 TABLE S1  Additional Data Set 1 contains the following information. The first sheet of this data set lists the complete OTU taxonomy for all 2,238 OTUs identified in the combined 160 samples from the main study individual. The second sheet of the data set lists OTUs that are categorized as either MGA (monotonous gradient ascending) or MGD (monotonous gradient descending). The top section of this sheet lists OTUs categorized as MGA (“lower GI preference”), while the bottom section of this data set lists OTUs categorized as MGD (“upper GI preference”). The third sheet lists OTUs observed having ascending or descending gradients in relative abundance with a breakpoint (GAB or GDB) at the ascending colon (AC). The fourth sheet of the data set lists OTUs observed having ascending or descending gradients in relative abundance with a breakpoint (GAB or GDB) at the transverse colon (TC). The fifth sheet of the data set lists OTUs observed having ascending or descending gradients in relative abundance with a breakpoint (GAB or GDB) at the descending colon (DC). The sixth sheet lists exact test results comparing the terminal ileum (TI) to all other regions. The seventh sheet lists exact test results comparing the ileocecal valve (IV) to all other regions. The eighth sheet lists exact test results comparing the ascending colon (AC) to all other regions. The ninth sheet lists exact test results comparing the rectum (R) to all other regions. The 10th sheet provides the ordered lists of genera and the corresponding OTUs for the heat maps relating to both the correlation analysis and the time series analysis. The 11th sheet lists the 136 most prevalent OTUs in the biopsy samples with the OTU number and the mean percent relative abundance in the biopsy samples. Download TABLE S1, XLSX file, 0.1 MB. Copyright © 2017 de Muinck et al. 2017 de Muinck et al. This content is distributed under the terms of the Creative Commons Attribution 4.0 International license.

**FIG 1:**
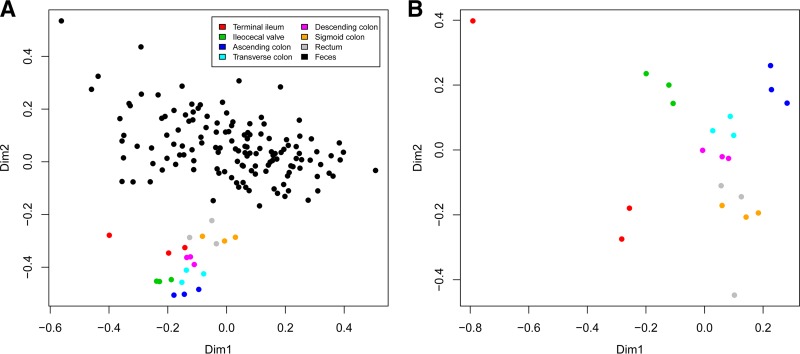
Nonmetric multidimensional scaling (NMDS) plots of fecal and biopsy sample-based Bray-Curtis distances computed from the relative abundances of the 258 most prevalent OTUs. (A) NMDS plot displaying fecal and biopsy samples from the main study individual. Colors represent the different sampling sites, and the distance between the circles represent similarity of the assemblage of OTUs comprising a sample. Clear separation was observed between the fecal samples and the biopsy samples (*P* << 0.001 by ANOSIM). Dim1, dimension 1. (B) NMDS plot of the biopsy samples obtained in triplicate from seven different locations along the GI tract. Significant structuring was observed between the anatomically distinct sampling sites (*P* << 0.001 by ANOSIM).

For the following analysis, we applied common scaling to the size of the smallest biopsy sample library (10,662 reads). OTUs were filtered so that it had to be observed in at least 0.1% relative abundance (11 reads) in at least three samples (equivalent to the number of replicate biopsy samples taken at an anatomically distinct site), leaving the 258 most prevalent OTUs. Within the main study individual, the biopsy samples obtained were clearly distinct from the fecal samples (Fig. 1A, *P* << 0.001 by analysis of similarity [ANOSIM]). Highly significant structuring (*P* << 0.001 by ANOSIM) was observed for the biopsy samples depending on sampling location, with the main axis of variation separating the TI from the AC (Fig. 1B). We repeated the analysis using OTU tables collapsed to the genus level in order to test the robustness of the structuring. Both for the data set including both fecal and biopsy samples and also for biopsy samples alone, the genus-level analysis resulted in Bray-Curtis distance matrices that correlated with the OTU-level distance matrices with Pearson coefficients of >0.96. In order to put our data in a broader context, the biopsy and fecal samples were compared with 15 fecal samples obtained sequentially from three healthy adults. These 15 samples formed three distinct clusters clearly separated from both the fecal and biopsy samples of the main subject (Fig. S1, *P* < 0.001 by ANOSIM).

10.1128/mSystems.00086-17.1 FIG S1  NMDS plot of the fecal and biopsy sample data of the main study individual (circles) compared with sequentially collected fecal samples from three healthy adult volunteers (triangles, squares, and crosses). Download FIG S1, EPS file, 0.01 MB. Copyright © 2017 de Muinck et al. 2017 de Muinck et al. This content is distributed under the terms of the Creative Commons Attribution 4.0 International license.

**FIG 2:**
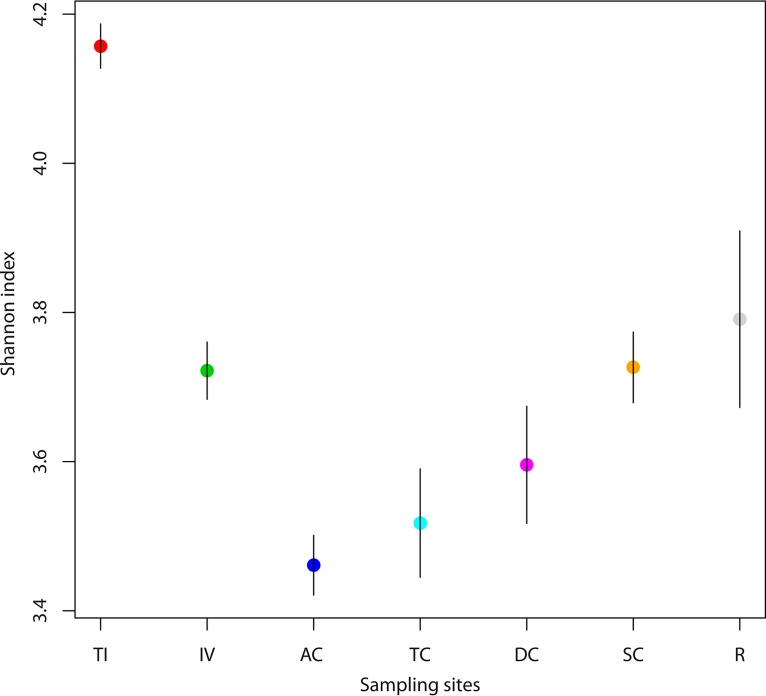
Bacterial diversity along the intestine as represented by the Shannon index. Bacterial diversity decreases from the terminal ileum to the ascending colon and then increases from the ascending colon until the rectum. Each circle represents the mean Shannon index for each sampling site, and gray bars represent the standard errors. The sampling locations are indicated on the *x* axis. Sampling site abbreviations: TI, terminal ileum; IV, ileocecal valve; AC, ascending colon; TC, transverse colon; DC, descending colon; SC, sigmoid colon; R, rectum.

At the phylum level, we observed an inverse relationship between the two most dominant phyla, *Bacteriodetes* and *Firmicutes*, with sigmoid-shaped patterns of mean relative abundances from the TI to the rectum (Fig. S2). The mean relative abundances for *Actinobacteria* decreased from the TI to the DC, and the mean relative abundances for *Tenericutes* increased from the TI to the rectum. We observed the highest OTU diversity (Shannon entropy) in the TI. The diversity dropped precipitously toward the AC and then increased steadily toward the rectum (Fig. 2). Interestingly, this pattern was less related to overall OTU richness (Fig. S3A) than it was to community evenness (Fig. S3B).

10.1128/mSystems.00086-17.2 FIG S2  Relative abundances of the six main bacterial phyla along the GI tract. The sampling locations are indicated on the *x* axis as follows: TI, terminal ileum; IV, ileocecal valve; AC, ascending colon; TC, transverse colon; DC, descending colon; SC, sigmoid colon; R, rectum. Means are indicated by circles that are color coded to conform to the other plots. Vertical lines indicate standard errors. Download FIG S2, EPS file, 0.01 MB. Copyright © 2017 de Muinck et al. 2017 de Muinck et al. This content is distributed under the terms of the Creative Commons Attribution 4.0 International license.

10.1128/mSystems.00086-17.3 FIG S3  (A) OTU richness along the GI tract. (B) Pielou’s evenness index along the GI tract. The sampling locations are indicated on the *x* axis as follows: TI, terminal ileum; IV, ileocecal valve; AC, ascending colon; TC, transverse colon; DC, descending colon; SC, sigmoid colon; R, rectum. Means are indicated by circles that are color coded to conform to the other plots. Vertical lines indicate standard errors. Download FIG S3, PDF file, 0 MB. Copyright © 2017 de Muinck et al. 2017 de Muinck et al. This content is distributed under the terms of the Creative Commons Attribution 4.0 International license.

### Spatial patterns along the intestinal tract.

**FIG 3:**
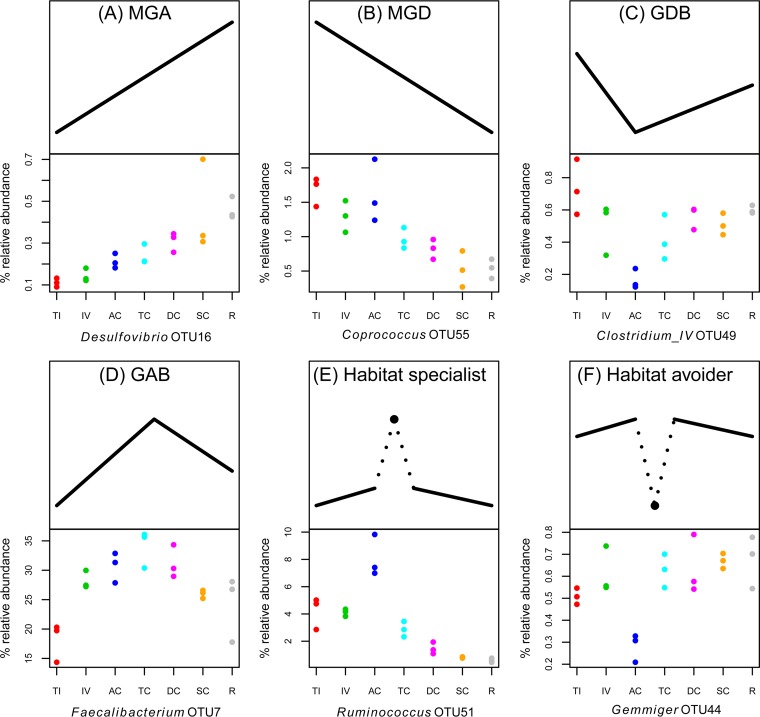
Examples of the six general categories of spatial structure observed along the GI tract. The top half of each panel illustrates the general patterns in relative abundance of a hypothetical OTU along the GI tract for each of the six different categories. (A) Monotonous gradient ascending (MGA). (B) Monotonous gradient descending (MGD). MGA and MGD refer to a significant positive or negative linear trend, respectively, in the relative abundances of an OTU along the intestinal tract, from the terminal ileum (TI) to the rectum (R). (C) Gradient descending with break point (GDB). (D) Gradient ascending with breakpoint (GAB). For GDB and GAB categorization, an OTU follows a “broken stick” pattern of relative abundances, where the linear regression coefficient changes sign in a segment along the GI tract. (E and F) Habitat specialists (HS) (E) and habitat avoiders (HA) (F). HS and HA refer to OTUs that have a significantly elevated or lowered relative abundance, respectively, in a particular segment of the intestine. The bottom half of each panel presents an example of an OTU identified in this study (along with a putative taxon assignment) that matches each of the observed categories of spatial patterning. The *x*-axis abbreviations are the same as those in Fig. 2. Note that using the above criteria, some OTUs can be placed into more than one category, e.g., OTU51 can be classified both as MGD and as HS (Fig. 3E).

The application of the above-described filtering procedure to the biopsy samples alone reduced the number of OTUs from 2,238 to the 136 most predominant OTUs (Table S1), accounting for an average of 93.5% (SD, 2.4%) of total per-sample reads. The distributions of these OTUs along the GI tract were relatively uniform (Fig. S4), but for several of the OTUs, we did observe significant spatial structure. We assigned these OTUs to six general categories (Fig. 3). For the monotonous gradient ascending (MGA) and monotonous gradient descending (MGD), we computed linear models under two basic assumptions. The GI tract can be considered a continuum or a series of physiologically distinct segments. In the former case, we regressed relative abundances of an OTU on the depth of sampling (centimeters from the anus), whereas in the latter, we assume equidistance between sampling sites by using the numbers 1 to 7 as the independent variable. Using both assumptions, we obtain similar results with 44 OTUs with significant (*P* < 0.05) trends under the first assumption and 59 OTUs with significant trends under the second assumption (Table S1). Similar numbers of OTUs were categorized as MGA (29 OTUs) and MGD (30 OTUs). Eighteen OTUs were classified as either gradient ascending with breakpoint (GAB) (5 OTUs) or gradient descending with breakpoint (GDB) (13 OTUs) with significant (*P* < 0.05) breakpoints in the AC (Table S1). We also observed lower numbers of OTUs with significant breakpoints in the TC (6 OTUs) and the DC (3 OTUs). We used exact tests for comparing one sampling site with all other sites in order to identify OTUs with significantly different occurrence (*P* < 0.01; false-discovery rate [FDR] < 0.05), and we categorized these as either habitat specialists (HS) or habitat avoiders (HA). We observed 36, 8, 36, and 2 OTUs with significantly different abundances in the TI, IV, AC, and rectum, respectively. No OTUs with significantly different abundances were observed in the TC, DC, or SC (Table S1).

10.1128/mSystems.00086-17.4 FIG S4  Relative abundances of the 63 main genera along the GI tract. Three samples were obtained from each of the regions along the GI tract, and each bar shows the mean relative abundance of the three replicates. The *x* axis lists the sampling sites as follows: TI, terminal ileum; IV, ileocecal valve; AC, ascending colon; TC, transverse colon; DC, descending colon; SC, sigmoid colon; R, rectum. OTU taxonomy assignments are shown in the accompanying color key. Download FIG S4, PDF file, 0.04 MB. Copyright © 2017 de Muinck et al. 2017 de Muinck et al. This content is distributed under the terms of the Creative Commons Attribution 4.0 International license.

### Cooccurrence modeling of the biopsy and fecal samples and time series analysis of the fecal samples.

We also analyzed 139 fecal samples obtained daily from the main study individual in order to identify interacting OTUs and relate these interactions to the spatial patterns observed in the biopsy sample data. We focused on the most prevalent and consistently observed OTUs by filtering the data such that an OTU had to constitute at least 0.05% of the reads (27 reads) in at least 90% of samples, reducing the number of OTUs from 2,238 to 76. The resulting data set represented an average of 78.3% (SD, 4.9%) of total per-sample reads, showing no major lasting shifts in community structure, and thus indicating that the data would be suitable for time series analysis (Fig. S5).

10.1128/mSystems.00086-17.5 FIG S5  Relative abundances of the 34 main genera observed in the fecal sample data, ordered by day (number of days) (*x* axis). Biopsy samples were obtained on day 75, indicated by the red vertical line on the plot. OTU taxonomy assignments are shown in the accompanying color key. Download FIG S5, PDF file, 0.1 MB. Copyright © 2017 de Muinck et al. 2017 de Muinck et al. This content is distributed under the terms of the Creative Commons Attribution 4.0 International license.

**FIG 4:**
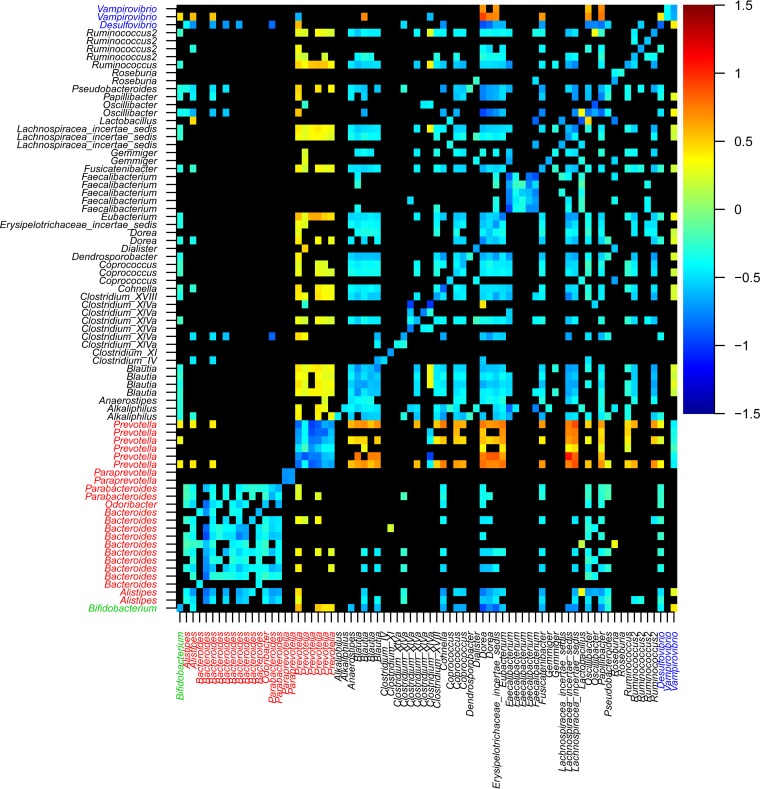
Bacterial interactions between 97% identified OTUs identified in the main study individual. The heat map shows the strength and direction of highly significant interactions after Benjamini-Hochberg correction for multiple testing (*P* < 0.01). Dependent variables are shown along the *y* axis, and independent variables are shown along the *x* axis, i.e., if you follow the column of a given OTU upward from the *x* axis until you reach a colored cell, that cell indicates the effect of the given OTU (independent) on the OTU indicated on the *y* axis (dependent). The color key on the right-hand side indicates the sign and magnitude of interactions that were significant. Cells representing nonsignificant relationships are black. Taxonomic assignments to the genus level are colored according to the phylum: green for *Actinobacteria*, red for *Bacteroidetes*, black for *Firmicutes*, and blue for *Proteobacteria*. Table S1 in the supplemental material lists the taxonomic assignments, in the order shown, with the matching OTU designations.

**FIG 5:**
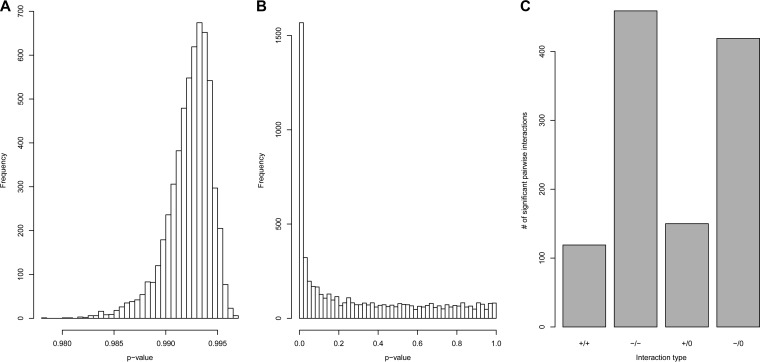
(A) Distribution of Benjamini-Hochberg-corrected *P* values resulting from recomputing the full set of time series models using randomly permutated dependent variables. (B) Distribution of Benjamini-Hochberg-corrected *P* values from the full set of original time series models. (C) Interaction categories from the time series analysis of the fecal sample data. The four categories are indicated on the *x* axis as follows: +/+ for cooperation, −/− for competition, +/0 for commensalism, and −/0 for amensalism. The *y* axis indicates the number of observed significant interactions in the specified categories.

We first computed a set of pairwise interaction models using the time series analysis approach (Fig. 4). Only 27% (1,345 of 5,700) of all the potential pairwise interactions were found to be significant at a 99% confidence level. Randomization of the order of the values in the independent variables in the time series models resulted in a set of *P* values normally distributed around 0.5. After applying the Benjamini-Hochberg correction for testing multiple hypotheses, not a single model resulting from variable randomization was found to be significant (Fig. 5A), as opposed to the original set of models with a distribution of *P* values that was highly skewed toward the low range (Fig. 5B). Of the interactions that were found to be significant, we observed four different categories of pairwise interaction (cooperation, competition, commensalism, and amensalism) in different proportions (Fig. 5C). The majority of the significant interactions were competitive, in line with previous observations (9, 14). Additionally, competition was more intense (i.e., regression coefficients were more strongly negative) for OTU pairs within a single phylum than between OTUs belonging to different phyla (mean coefficients of −0.2 versus 0.01; *P* << 0.001 by one-sided Wilcoxon’s rank sum test). This observation was even more pronounced when comparing within-genus interaction to interactions between OTUs belonging to different genera (mean coefficients of −0.29 versus −0.08; *P* << 0.001 by one-sided Wilcoxon’s rank sum test).

**FIG 6:**
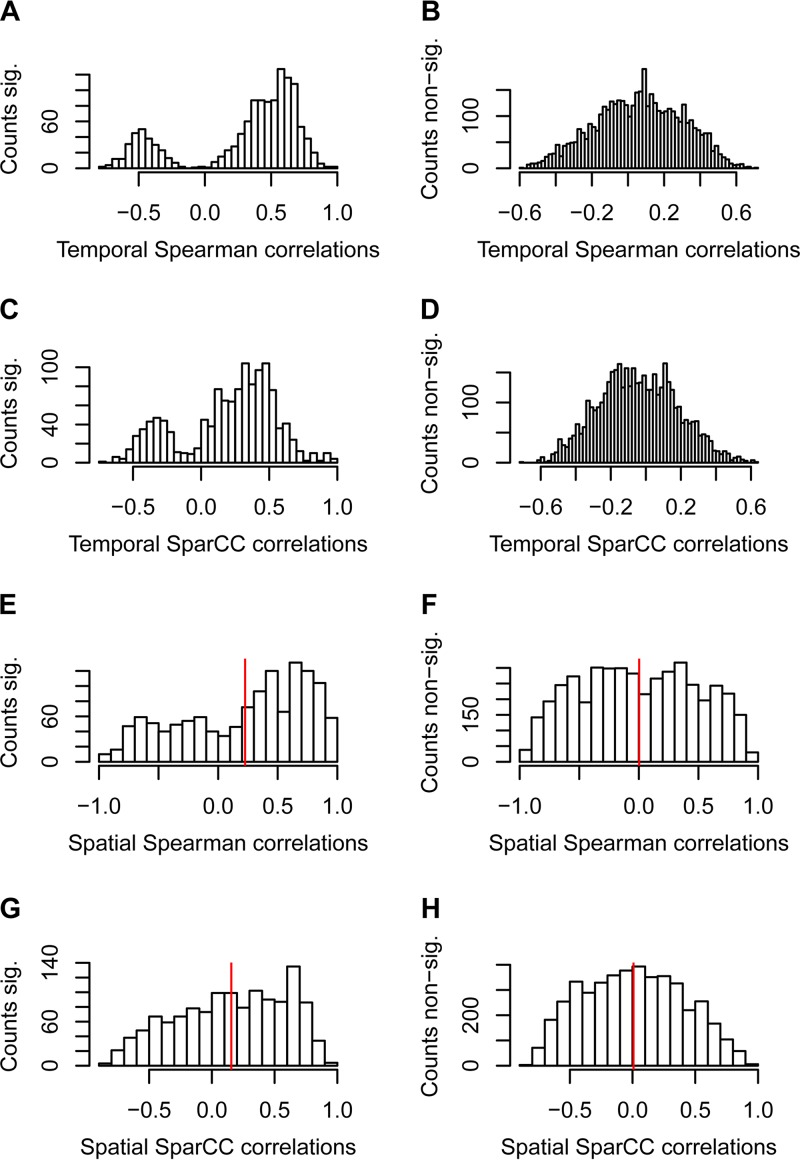
Distributions of pairwise correlation coefficients from cooccurrence models computed from temporal data (fecal) and spatial data (biopsy samples; means of three replicates). For each pair of panels, correlation coefficients were partitioned into significant (A, C, E, and G) or nonsignificant (B, D, F, and H) based on the significance values from the full set of time series models. (A and B) Temporal Spearman correlations; (C and D) temporal SparCC correlations; (E and F) spatial Spearman correlations; (G and H) spatial SparCC correlations. The vertical red lines in panels E to H represent the distribution means. Note that panels E and G have a significant positive skew (means of 0.22 and 0.16, respectively; *P* << 0.001 for both tests), indicating correspondence between the spatial and temporal correlation patterns.

In order to compare the methodologies, we also computed a set of pairwise interaction models using a cooccurrence approach based on contemporaneous correlations using both Spearman correlations between relative OTU abundances as well as the SparCC algorithm for computing Pearson correlations between transformed OTU counts (15) (Fig. S6). There was strong agreement between the time series modeling approach and both cooccurrence modeling techniques, with highly significant negative correlations between the regression coefficients from the time series models and the correlation coefficients from both the Spearman and SparCC approach (Spearman’s rho = −0.85 and Pearson’s *r* = −0.70, respectively; *P* << 0.001 for both comparisons). Interestingly, the correlation coefficients corresponding to significant time series models follow a strictly bimodal distribution in which the interactions discovered from the time series modeling that are negative are associated with positive contemporaneous correlations, while positive interactions are associated with negative correlations (Fig. 6A and C), while nonsignificant models follow a roughly normal distribution centered around zero (Fig. 6B and D).

10.1128/mSystems.00086-17.6 FIG S6  Pairwise Spearman (A) and SparCC (B) correlations between pairs of OTUs (97% identified) in the fecal sample data from the main study individual. The heat map describes the strength of correlations as indicated in the color key on the right-hand side (correlations <0.25 are blacked out). The *y*-axis and *x*-axis labels list the genus-level taxon assignments of OTUs. Taxon names are colored according to phylum: green for *Actinobacteria*, red for *Bacteroidetes*, black for *Firmicutes*, and blue for *Proteobacteria*. Table S1 lists the genera, in the order shown, with the matching OTU designations. Download FIG S6, PDF file, 0.1 MB. Copyright © 2017 de Muinck et al. 2017 de Muinck et al. This content is distributed under the terms of the Creative Commons Attribution 4.0 International license.

We then compared the time series models with cooccurrence models based on spatial correlations between the same 76 OTUs in the biopsy samples (Fig. S7). In order to check for agreement between the modeling approaches, we again compared the distribution of spatial correlation coefficients corresponding to significant pairwise time series models to the distribution of spatial correlation coefficients corresponding to nonsignificant time series models. If there were agreement between the two approaches, we should, as above, observe a significant positive skew in the distribution of the correlation coefficients corresponding to significant time series models, while for the nonsignificant models, the distribution should be centered around zero. This is based on the rationale that interactions are predominantly negative due to resource competition, which would result in a predominance of positive cooccurrence patterns along the intestinal tract. We observed a highly significant positive skew for both the Spearman (means of 0.22 and 0.002 for correlation coefficients corresponding to significant and nonsignificant time series models, respectively; *P* << 0.001 by Wilcoxon’s rank sum test; Fig. 6E and F) and SparCC models (means of 0.156 and 0.008, respectively; *P* << 0.001; Fig. 6G and H), indicating competing taxa that are actually colocalized and interacting. As a further comparison, we also checked for agreement between cooccurrence models as based either on spatial (biopsy samples) or contemporaneous (fecal samples) correlations as well as correspondence between spatial correlations and interaction coefficients from time series modeling. We observed noisy but significant positive correlations between contemporaneous and spatial correlation coefficients both when using the Spearman approach (*r* = 0.29 and *P* << 0.001 by Pearson’s correlation test; Fig. S8A) and the SparCC approach (*r* = 0.31 and *P* << 0.001; Fig. S8C). Comparison of spatial correlations with time series modeling coefficients gave similar results (*r* = −0.28 and −0.21 for Spearman and SparCC models, respectively, *P* << 0.001 for both tests, Fig. S8B and D), but with the expected negative relationship, signifying a preponderance of negative interactions.

10.1128/mSystems.00086-17.7 FIG S7  Pairwise Spearman (A) and SparCC (B) correlations between pairs of OTUs (97% identified) based on the mean OTU relative abundances from triplicate biopsy samples. The heat map shows the strength of correlations as indicated in the color key on the right-hand side (correlations <0.25 are blacked out). The *y-*axis and *x*-axis labels list the genus-level taxon assignments of OTUs. Taxon names are colored according to phylum: green for *Actinobacteria*, red for *Bacteroidetes*, black for *Firmicutes*, and blue for *Proteobacteria*. Table S1 lists the genera, in the order shown, with the matching OTU designations. Download FIG S7, PDF file, 0.1 MB. Copyright © 2017 de Muinck et al. 2017 de Muinck et al. This content is distributed under the terms of the Creative Commons Attribution 4.0 International license.

10.1128/mSystems.00086-17.8 FIG S8  (A) Relationship between the Spearman correlation coefficients between OTU pairs in the temporal (fecal) data and the spatial Spearman correlation coefficients between OTU pairs in the biopsy samples (*r* = 0.29, *P* << 0.001, Pearson’s correlation test). (B) Relationship between the regression coefficients from the time series models describing pairwise OTU interactions and the spatial Spearman correlation coefficients between OTU pairs in the biopsy samples (*r* = −0.28, *P* << 0.001). (C) Relationship between the SparCC correlation coefficients between OTU pairs in the temporal (fecal) data and the spatial SparCC correlation coefficients between OTU pairs in the biopsy samples (*r* = 0.31, *P* << 0.001, Pearson’s correlation test). (D) Relationship between the regression coefficients from the time series models describing pairwise OTU interactions and the spatial SparCC correlation coefficients between OTU pairs in the biopsy samples (*r* = −0.21, *P* << 0.001). Download FIG S8, PDF file, 0.1 MB. Copyright © 2017 de Muinck et al. 2017 de Muinck et al. This content is distributed under the terms of the Creative Commons Attribution 4.0 International license.

## DISCUSSION

In this study, different DNA extraction methods were employed for the two sample types due to the limited amount of sample material in the biopsy samples (low DNA yield) and different requirements for purification (i.e., high levels of potential inhibitors to downstream PCR in the fecal samples). DNA extraction procedures can affect the observed distribution of OTUs, which is a known source of bias in microbiome studies (16, 17), although some reports have found this issue to be less serious (18, 19). Even though DNA extraction techniques differed between the biopsy samples and the fecal samples, we observed that, within an individual, the mucosa-associated microbiota clustered according to sampling site and seemed to cluster more closely with the feces as the distance from the anus decreased (Fig. 1A). Furthermore, we observed clustering by individual rather than by DNA extraction method (see Fig. S1 in the supplemental material). This is in line with what others have observed in that GI microbiomes are highly individual (4, 8).

There have been conflicting reports of differences in microbiota diversity along the GI tract (20, 21). However, we sampled a larger number of sites from the small intestine to the rectum. We report a very clear pattern with the highest diversity in the terminal ileum (TI), the lowest in the ascending colon (AC), and a gradual increase toward the rectum (Fig. 2). This is similar to the model postulated by Donaldson et al. (2), of higher diversity toward distal sections due to a decreasing gradient of antimicrobials toward the rectum. These structural patterns, as well as the inverse relationship between the relative abundances of *Bacteriodetes* and *Firmicutes* (Fig. S2), the two most dominant phyla, lend evidence to the notion that different segments of the intestine can promote distinct microbial community structures. We do not have a strong explanation for the reduced diversity observed in biopsy samples from the AC, but one possible hypothesis is that the AC represents a type of filter for promoting separation of the bacterial communities associated with the small intestine and the colon. This might provide a mechanism for the host to compartmentalize the functions provided by the GI microbiota and may be related to the observation that OTU richness was relatively stable throughout the GI tract, while evenness increased from the AC to the rectum (Fig. S3).

Overall, in the biopsy samples, we found several OTUs that we could place into six different general categories of spatial occurrence. In addition to monotonous gradients in relative abundance (monotonous gradient ascending [MGA] and monotonous gradient descending [MGD]), which have been observed by others (2), we also observed several instances of “broken stick” patterns (gradient descending with breakpoint [GDB] and gradient ascending with breakpoint [GAB]), as well as OTUs with significantly higher or lower relative abundances in certain segments (habitat specialists [HS] or habitat avoiders [HA]). The OTUs that were found to have monotonous gradients in relative abundance along the intestine may be explained by the physiochemical gradients in pH, oxygen, nutrients, and antimicrobials, etc. (2), the categories GDB, GAB, HS, and HA suggest that other factors may be involved. However, there is little available information about the fine-scale environmental conditions along the GI tract. From a statistical standpoint, one would have more power to detect significant breakpoints in the transverse colon (TC), that being the midpoint of spatial sampling. However, we found that the majority of breakpoints were in the AC, suggesting that the environmental conditions in this compartment may strongly modulate the microbial community. This observation is also reflected in the diversity estimates of the AC.

Several of the most predominant OTUs displayed significant spatial structure along the GI tract. The most represented OTU in the combined data set (OTU1, 100% BLAST identity to *Prevotella copri*) was significantly (linear model *P* < 0.01) more prevalent in the upper GI tract. The most dominant taxa in the biopsy samples (OTU7, 99% BLAST identity to *Faecalibacterium prausnitzii*), was a GAB with an AC breakpoint. Interestingly, *Sutterella* (OTU43, 100% BLAST identity to *Suterella* sp. strain Marseille-P2435 [accession no. LT223579]) and *Ruminococcus* (OTU51, 100% BLAST identity to *Ruminococcus faecis* strain Eg2 [accession no. NR_116747.1]) are both categorized as GAB with the breakpoint in the ascending colon. Both *Ruminococcus* and *Sutterella* GI tract colonization have been linked to autism spectrum disorder (22, 23). Another *Ruminococcus* (OTU27, 99% BLAST identity to *Ruminococcus bromii* strain ATCC 27255, NR_025930.1) was GDB with the breakpoint at the AC. This species has been described as a potential “keystone species” for resistant starch degradation in the human intestine (24).

Cooccurrence modeling is based on the rationale that negatively and positively correlated occurrence patterns arise from negative and positive interactions, respectively (25). In our analyses, we actually observed the opposite pattern, i.e., negatively interacting OTUs, as estimated by time series analysis, displayed positive contemporaneous correlations and vice versa. This result was robust to the algorithm used for computing correlations, and it was also reflected in the spatial correlation patterns observed for the biopsy samples. We do not have a definite explanation for these observations, but we offer the following hypothesis. If a pair of taxa have a preference for the same resource, they are likely to be found at locations where that resource is available, and thus have a positive cooccurrence pattern. On the other hand, they would have to compete for said resource, which would explain the preponderance of negative regression coefficients in the time series models. This hypothesis also favors habitat filtering rather than species assortment as the main community-level assembly rule, which is in agreement with previous research (26).

As observed in previous work (9), negative interactions were more intense between more closely related OTUs. This notion goes all the way back to Darwin (27) and forms the basis of the principle known as “reverse ecology” which uses metabolic overlap deduced from genome sequences to infer ecological interaction (26). This matches in particular the high levels of competition that we observed within the genera *Bacteroides* and *Parabacteroides*. Both the time series and cooccurrence modeling identified large numbers of positive interactions involving OTUs classified as *Prevotella* and OTUs from other genera, but the *Prevotella* had relatively few significant interactions with OTUs classified as *Bacteroides*. *Prevotella* spp. have been shown to often have a negative contemporaneous correlation in relative abundance with *Bacteroides* (28–30). We also made this observation. Taken together, these results suggest that the interaction between *Prevotella* and *Bacteroides* is not in fact directly antagonistic but is instead mostly mediated by other factors such as diet, an interpretation which is in concordance with work suggesting that certain dietary components enhance *Prevotella* abundance (12, 29, 31). An OTU had *Bdellovibrio* spp. (85% BLAST identity to *Vampirovibrio chlorellavorus* strain ICPB 3707), a predatory bacterium, as its closest known relatives. While negatively affecting *Prevotella*, this bacterium is positively affected by several other genera, supporting the classification as a gut predatory bacterium from an ecological basis.

Microbiome studies of the GI tract are almost commonplace. 16S rRNA gene amplicon sequencing provides a limited view of complex bacterial communities. However, there remain many unanswered questions that these types of surveys are well equipped to address. Here we present data showing clear spatial structure in the microbiota associated with distinct regions of the GI tract. We also identified OTUs that conformed to six general categories of spatial occurrence patterns. Finally, we have demonstrated that the spatial structure of the microbiota in the GI tract relates to the interaction structure inferred from the time series analysis of fecal samples. In a previous study (4), one fecal sample from each of three subjects was obtained at a time point 3 months after the biopsy samples were taken in order to lessen the potential influence of the colonic cleansing on the GI microbiota. Interestingly, we performed the biopsy sampling during an approximate midpoint in the sampling period and observed no marked effect of bowel cleansing on the overall structuring of the microbiota (Fig. S5). We also observed significant general agreement between the interaction models derived from analyzing either biopsy samples or longitudinal fecal data, lending some support to the use of fecal samples to gain an understanding of the adherent microbiota. The strong accordance between cooccurrence and time series modeling approaches to the fecal data suggests that both approaches are valid for identifying biotic interactions. The time series approach has the benefit of being able to describe directionality in nonsymmetric interactions. However, combining both techniques can facilitate the discovery of interactions that are not directly antagonistic or facilitative but that are mediated by environmental factors, as may be the case of *Bacteriodetes* and *Prevotella* in this study.

## MATERIALS AND METHODS

### Sampling procedures.

The study obtained ethical approval from the regional ethical committee of Norway, and the participants gave signed informed consent. The subjects were not taking any medication during the course of the study. The main study individual had not used antibiotics within the past 2 years or during the study; this individual underwent standard bowel cleansing with Fleet Phospho-Soda the evening before the colonoscopy. Endoscopically, the colonic mucosa appeared grossly normal. Colonic tissue biopsy samples (approximately 1 by 2 mm each) were collected in triplicate from the terminal ileum (TI; about 155 cm from the anus), ileocecal valve (IV; about 150 cm from the anus), ascending colon (AC; about 142 cm from the anus), transverse colon (TC; about 109 cm from the anus), descending colon (DC; about 64 cm from the anus), sigmoid colon (SC; about 20 cm from the anus), and rectum (about 10 cm from the anus) during colonoscopy. Samples were then placed in cryovials and snap-frozen in liquid nitrogen. The cryovials were transferred to an −80°C freezer for storage until further processing. Fecal samples were frozen immediately upon collection at −20°C and then transferred to −80°C until further processing.

### DNA extraction.

The biopsy samples were transferred to FastPrep tubes with 210 µl of 20% SDS, 500 µl phenol-chloroform-isoamyl alcohol (25:24:1), 500 µl H_2_O, and ~200 mg acid-washed glass beads (≤106 µm) (Sigma-Aldrich). The FastPrep tubes were then shaken at 4 m/s for 1 min (FastPrep-24; MP Biomedicals) and centrifuged at 13,200 rpm for 5 min, and ~600 µl of the aqueous phase was transferred to a clean 1.5-ml microtube. Sixty microliters of ice-cold 3 M sodium acetate and 450 µl ice-cold isopropanol were added, and the solution was mixed. The tubes were then incubated at −20°C for 30 min and centrifuged at 13,200 rpm at 4°C for 15 min. The supernatant was then removed, and the pellet was washed with 1 ml ice-cold ethanol (EtOH) before resuspension in 20 µl buffer AE (Qiagen). An additional cleaning step was then carried out using molecular biology-grade glycogen (Thermo Fisher, Waltham, MA, USA) according to the manufacturer’s instructions. Approximately 240 mg of fecal material was used for DNA extraction with the PowerSoil 96-well DNA isolation kit (Mo Bio Laboratories, Inc., Carlsbad, CA, USA).

### Illumina sequencing and data processing.

Library preparation for Illumina sequencing was conducted by the procedure of de Muinck et al. (13). Sequencing was done on an Illumina HiSeq 2500 apparatus (Illumina, San Diego, CA, USA) using the 250-bp paired-end rapid-run mode. Low-quality reads were trimmed and Illumina adapters were removed using Trimmomatic v0.36 (32) with default settings. Reads mapping to the PhiX genome (NCBI identifier [ID] or accession no. NC_001422.1) were removed using BBMap v36.02 (33). Demultiplexing of data based on the dual index sequences was performed using custom scripts available at github (https://github.com/arvindsundaram/triple_index-demultiplexing). Internal barcodes and spacers were removed using cutadapt v1.4.1 (34), and paired reads were merged using FLASH v1.2.11 (35) with default settings. Sequence files were then converted from fastq to fasta, and primers were trimmed from merged read ends. Further processing of sequence data was conducted using a combination of vsearch v2.0.3 (36) and usearch v8.1.1861 (37). Specifically, dereplication was performed with the “derep_fulllength” function in vsearch with the minimum unique group size set at 2. Operational taxonomic unit (OTU) clustering, chimera removal, taxonomic assignment, and OTU table building were carried out using the uparse pipeline (38) in usearch. Taxonomic assignment to the genus level was done against the RDP-15 training set. Samples from the main study individual were clustered as a single data set so that a given OTU number from a biopsy sample corresponds to the same OTU number in a fecal sample. Clustering was redone when including additional adult samples.

### Statistical analyses.

Read depths were normalized by common scaling (39). This entails multiplying each OTU count in a given library with the ratio of the smallest library size to the size of the individual library. This procedure replaces rarefying (i.e., random subsampling to the lowest number of reads), as it produces the library scaling one would achieve by averaging over an infinite number of repeated subsamplings. After library scaling, the data were filtered according to the criteria stated below or in each section of the results. All statistical analyses were done using R (40). Bray-Curtis distance matrix computation and analyses of similarity (ANOSIMs, 10,000 permutations) were carried out using the “vegan” package. Nonmetric multidimensional scaling (NMDS) was carried out using the “isoMDS” function in the MASS package. Exact tests for differences in means between two groups of negative binomially distributed counts (41–43) were performed using the edgeR package (44). Although originally developed for analysis of differential expression in RNA sequencing experiments, this method has been shown to perform excellently in identifying enriched OTUs in 16S rRNA gene amplicon sequencing experiments as well (39). For these tests, we did not use common scaled data, as library size differences are accounted for as part of the statistical algorithm. Specific filtering criteria were used for exact tests in order to focus on the most abundant OTUs. For an OTU to be included in this analysis, it had to be observed at a relative abundance of at least 0.1% in at least three samples. This filtering procedure reduced the number of OTUs analyzed from 2,238 to 136. For the diversity measures, we did not perform filtering of the OTU tables other than library size scaling and singleton removal. This procedure removed any significant relationship between library size and observed OTU diversity. Time series modeling was performed as described in reference 9. Briefly, the dynamics of each OTU was modeled according to the equation, *x*_i,*t+1*_* − x*_i,*t*_* =* α_*i*,*j*_* +* β_*i*,*j*_*x*_j,*t*_, where *x*_*i*_,*t* is the log relative abundance of taxon *i* at time *t*, α_*i*_,_*j*_ are intercept terms, β_*i*_,_*j*_ are linear regression coefficients, and *x*_j,_*t* are log relative abundances of taxon *j* at time *t*. The regression coefficients are then interpreted as describing the biotic interaction between OTUs *i* and *j*. The strength of the interaction is proportional to the size of the coefficient, with a positive coefficient signifying a cooperative or commensal interaction and a negative coefficient signifying competition or amensalism. Each element in the resulting interaction matrix is estimated from separate models (i.e., variable A as a function of variable B or B as a function of A) with different dependent and independent variables, resulting in a nonsymmetric matrix. This allows for identification of nonreciprocal interactions (commensalism or amensalism) or pairwise interactions with opposite signs (parasitism), as well as reciprocal pairwise relationships (cooperation and competition). The estimated within-OTU interactions (diagonal of the interaction matrix) should be interpreted with caution, as the independent variable term is also part of the dependent variable, and these coefficients are excluded from further analysis. The total number of equations in a system is equal to *n*^2^, where *n* is the total number of taxa. This approach does not capture relationships that are strongly nonlinear that could be modeled, e.g., by generalized additive models, but previous work has shown linear regression to be a good approximation (11). Time series models were computed only for the most abundant OTUs (observed in at least 90% of samples), since the modeling technique is effective only for OTUs observed consistently and at levels that provide good estimations of relative abundance. Model *P* values were corrected for multiple hypotheses tested by applying the Benjamini-Hochberg adjustment in order to reduce the false-discovery rate (45). In order to test the robustness of the time series approach, the full set of models was recomputed using random permutations of the dependent term (*x*_j,*t*_) 100 times, and *P* values were averaged over the replicated matrices. Cooccurrence modeling was conducted using two alternative methodologies. First, we computed pairwise Spearman correlation coefficients between relative OTU abundance profiles (25). Since studies have shown that simple Spearman correlations can produce spurious results when analyzing microbiome data (15), we also apply a more sophisticated approach. This is known as SparCC (15) and is designed for large, sparse, abundance matrices of the kind obtained in microbiome studies. We used the “sparcc” implementation in the mothur software suite (46), with default settings, in order to obtain a more robust estimation of the cooccurrence matrix. We use both of these cooccurrence modeling approaches to compute contemporaneous and spatial correlations. Contemporaneous correlations were computed for the longitudinally obtained fecal samples and provide information about which OTUs tend to cooccur over time. Spatial correlations were computed for the biopsy samples and provide information about which OTUs tend to reside in the same locations along the GI tract. In contrast to the time series modeling approach, correlation mapping forces a symmetrical pairwise interaction matrix and thus cannot identify nonreciprocal interactions or interactions with opposite signs.

### Accession number(s).

All sequence data used in this study have been made available at the NBCI Sequence Read Archive under the BioProject ID PRJNA387407.
