# Supplementary figures and images for: Linking Spatial Structure and Community-Level Biotic Interactions through Cooccurrence and Time Series Modeling of the Human Intestinal Microbiota
Source: mSystems. 2017 Sep 5;2(5):e00086-17. doi: 10.1128/mSystems.00086-17 (PMC5585691; doi:10.1128/mSystems.00086-17)

**A**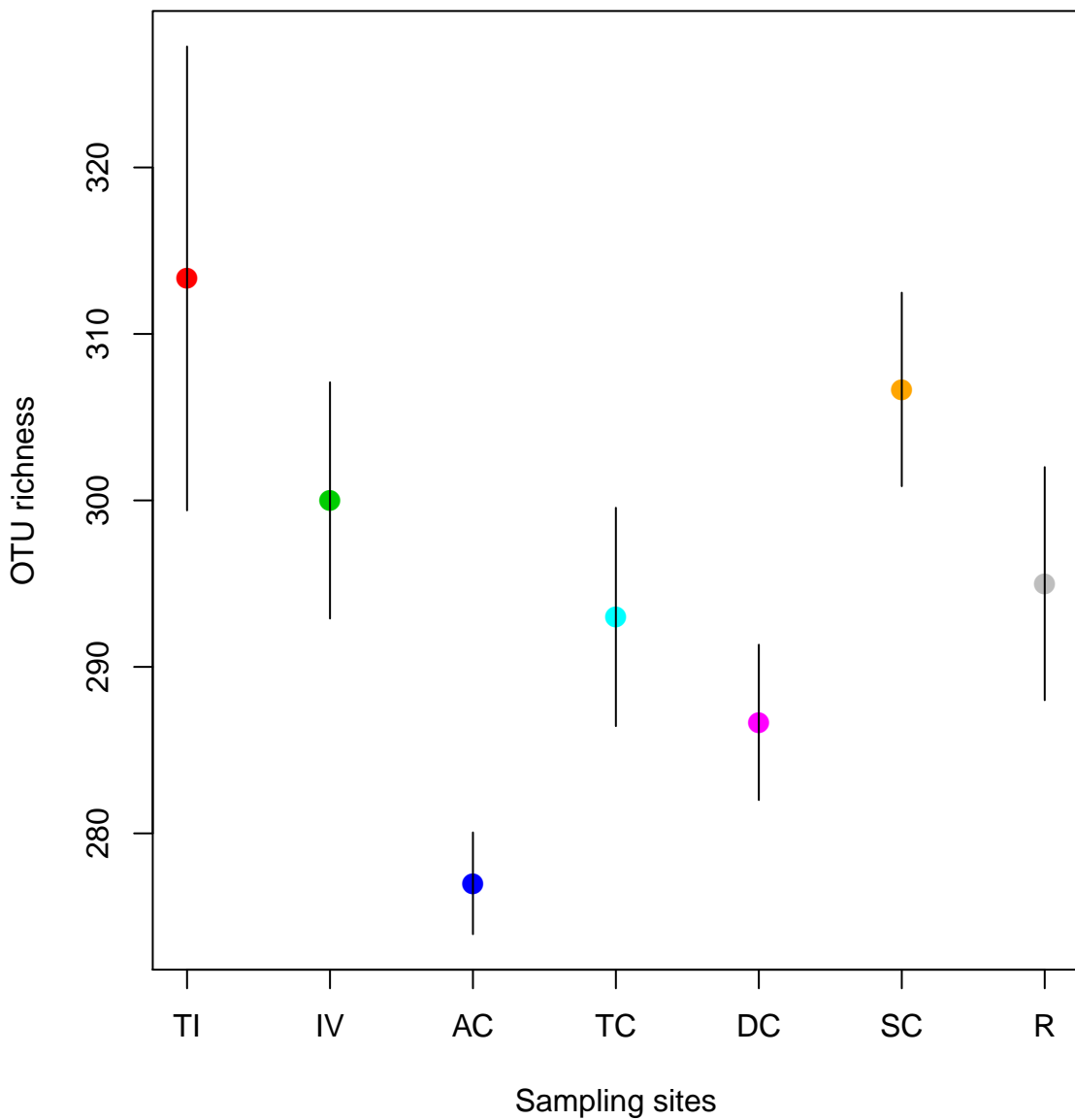**B**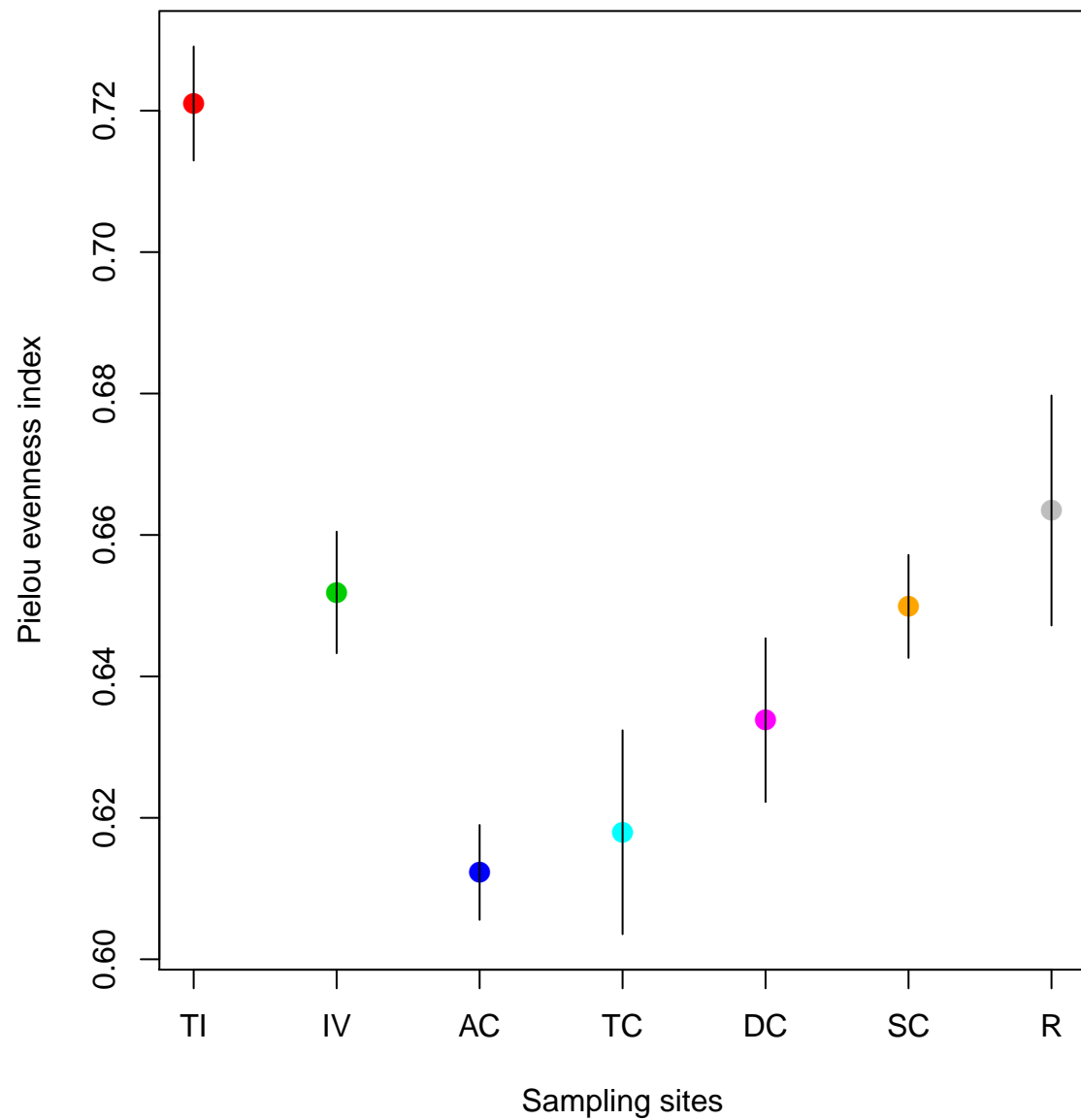

Supplement: FIG S3 [file sys001162135sf3.pdf]

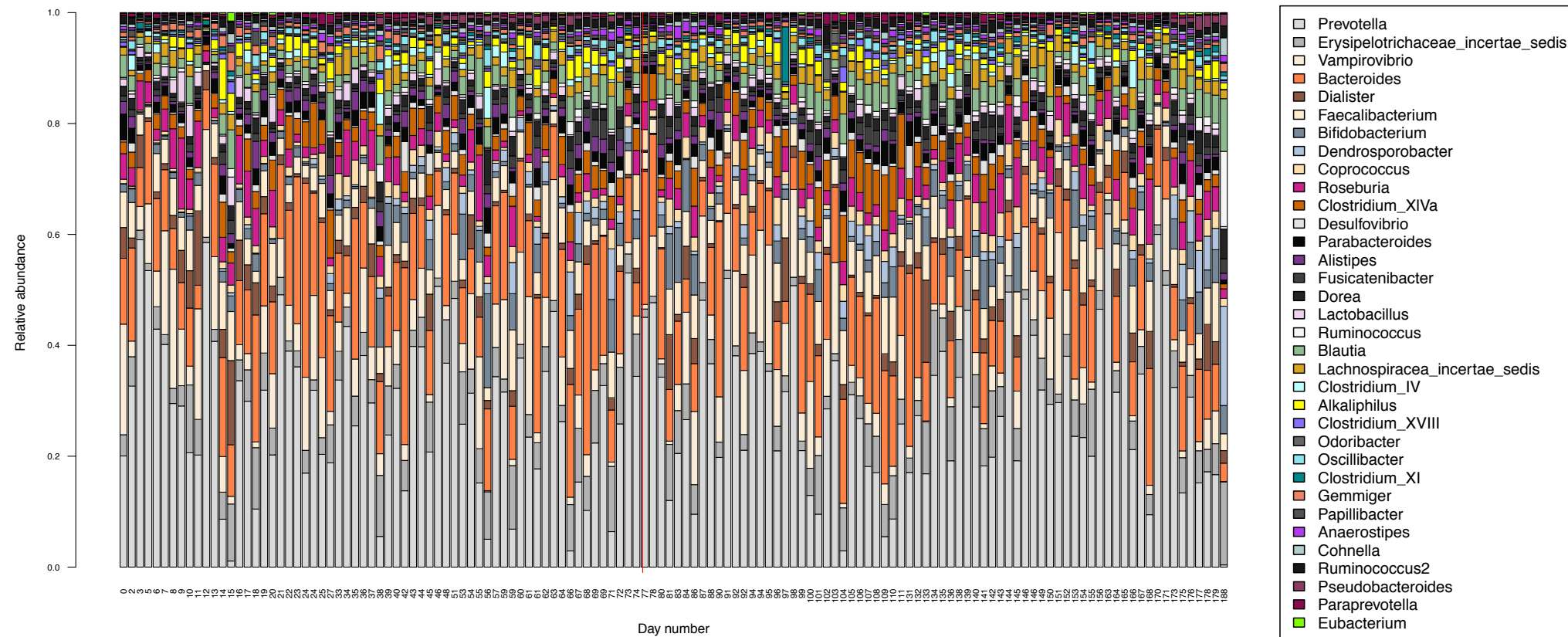

Supplement: FIG S5 [file sys001162135sf5.pdf]

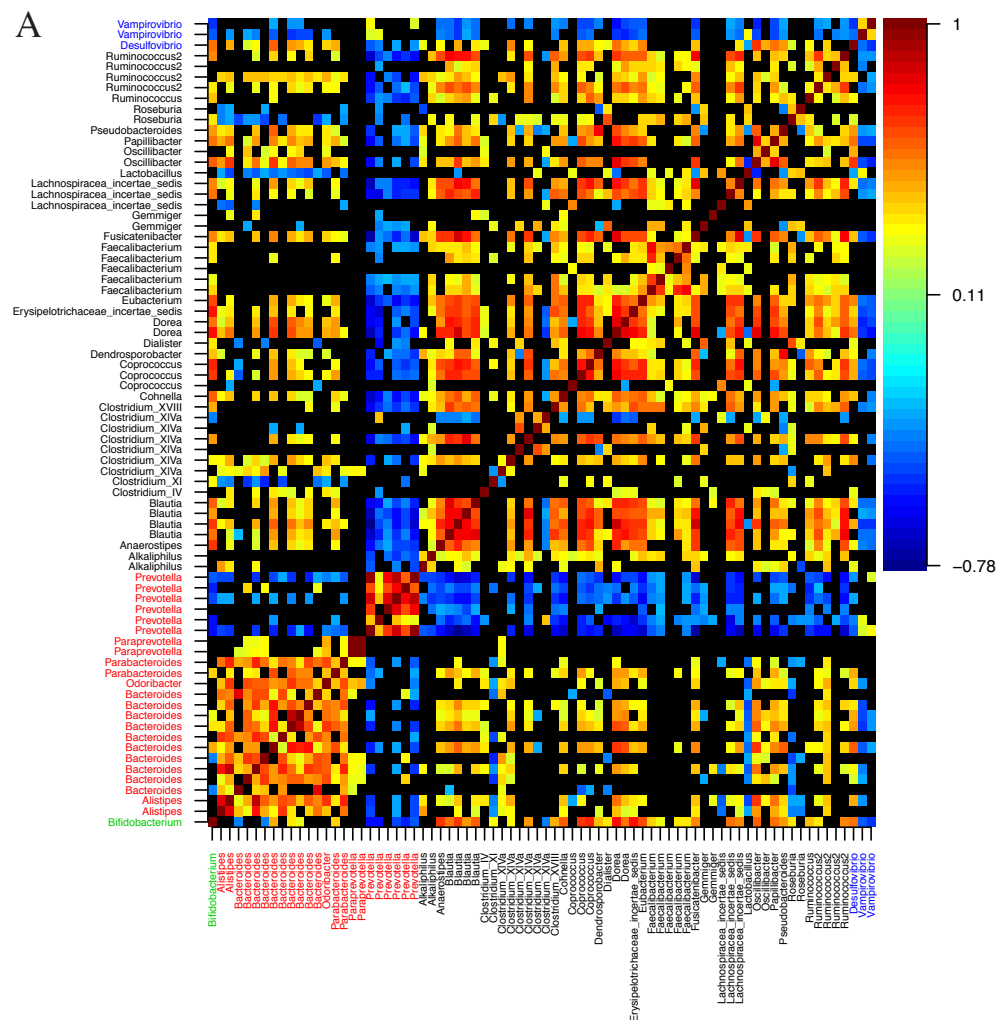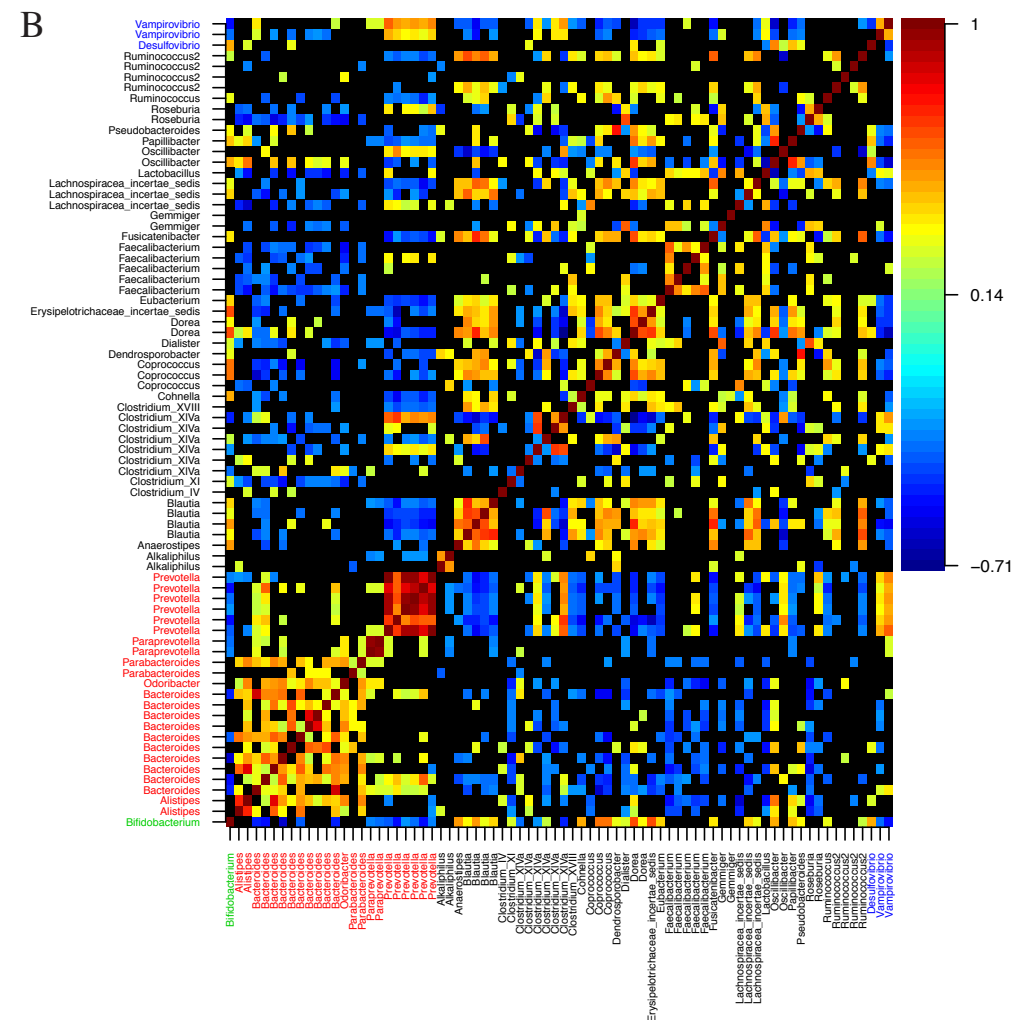

Supplement: FIG S6 [file sys001162135sf6.pdf]

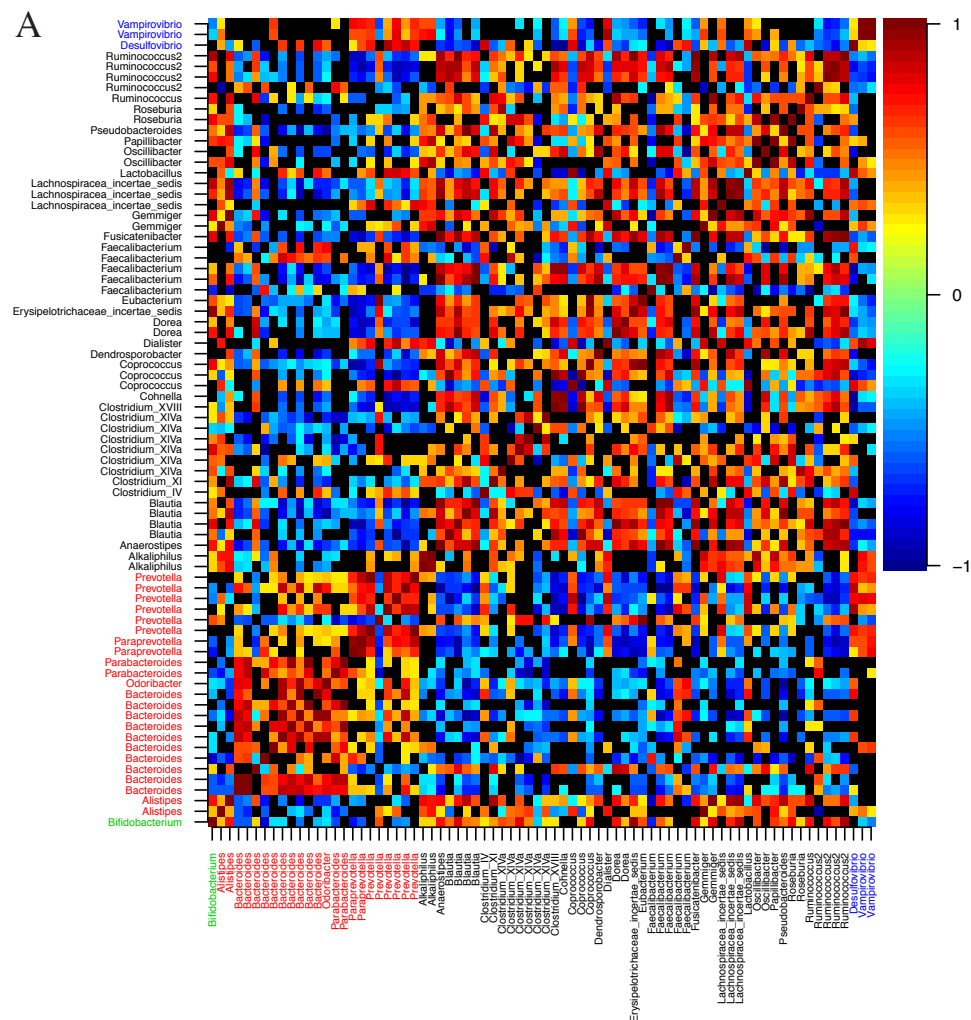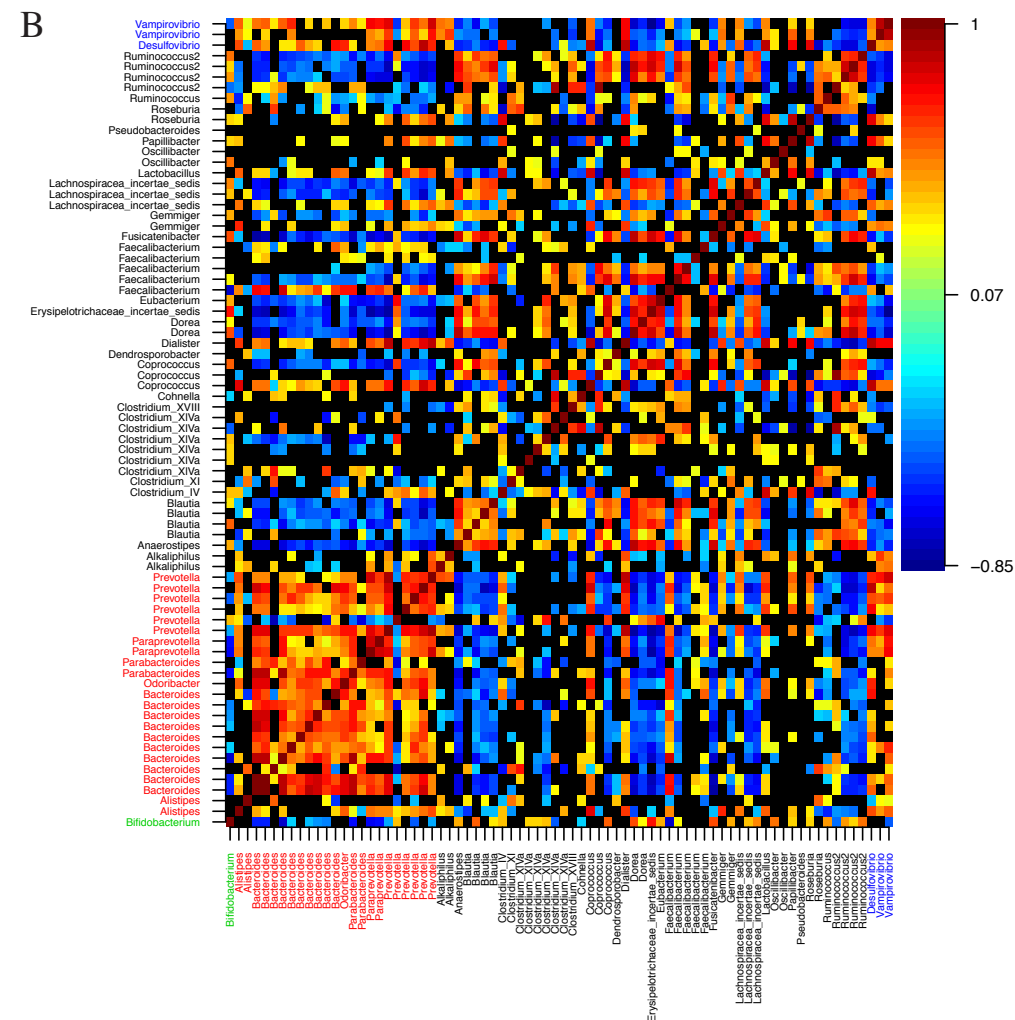

Supplement: FIG S7 [file sys001162135sf7.pdf]

**A**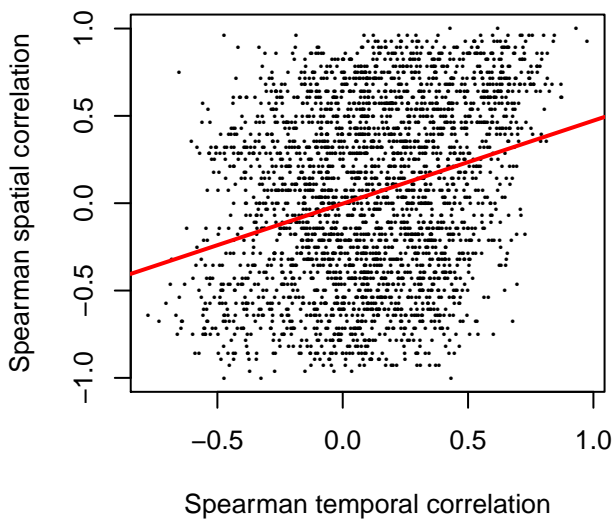**B**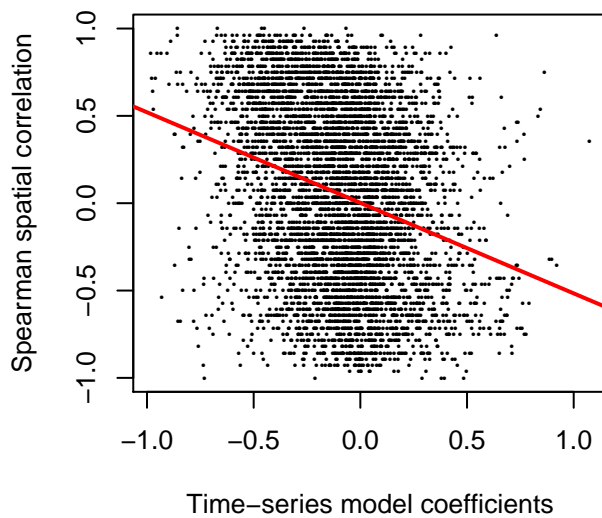**C**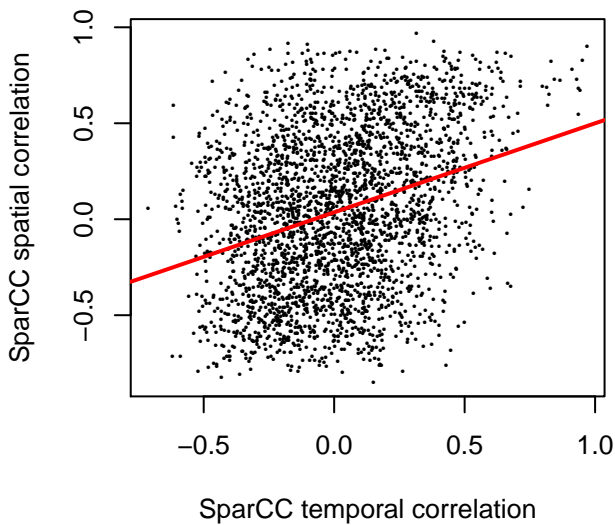**D**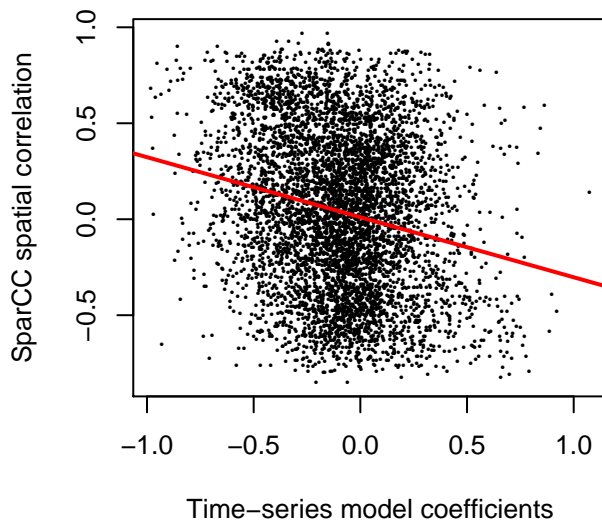

Supplement: FIG S8 [file sys001162135sf8.pdf]
