# Supplementary material for: Linking Spatial Structure and Community-Level Biotic Interactions through Cooccurrence and Time Series Modeling of the Human Intestinal Microbiota
Source: mSystems. 2017 Sep 5;2(5):e00086-17. doi: 10.1128/mSystems.00086-17 (PMC5585691; doi:10.1128/mSystems.00086-17)
Supplement: FIG S4 [file sys001162135sf4.pdf]

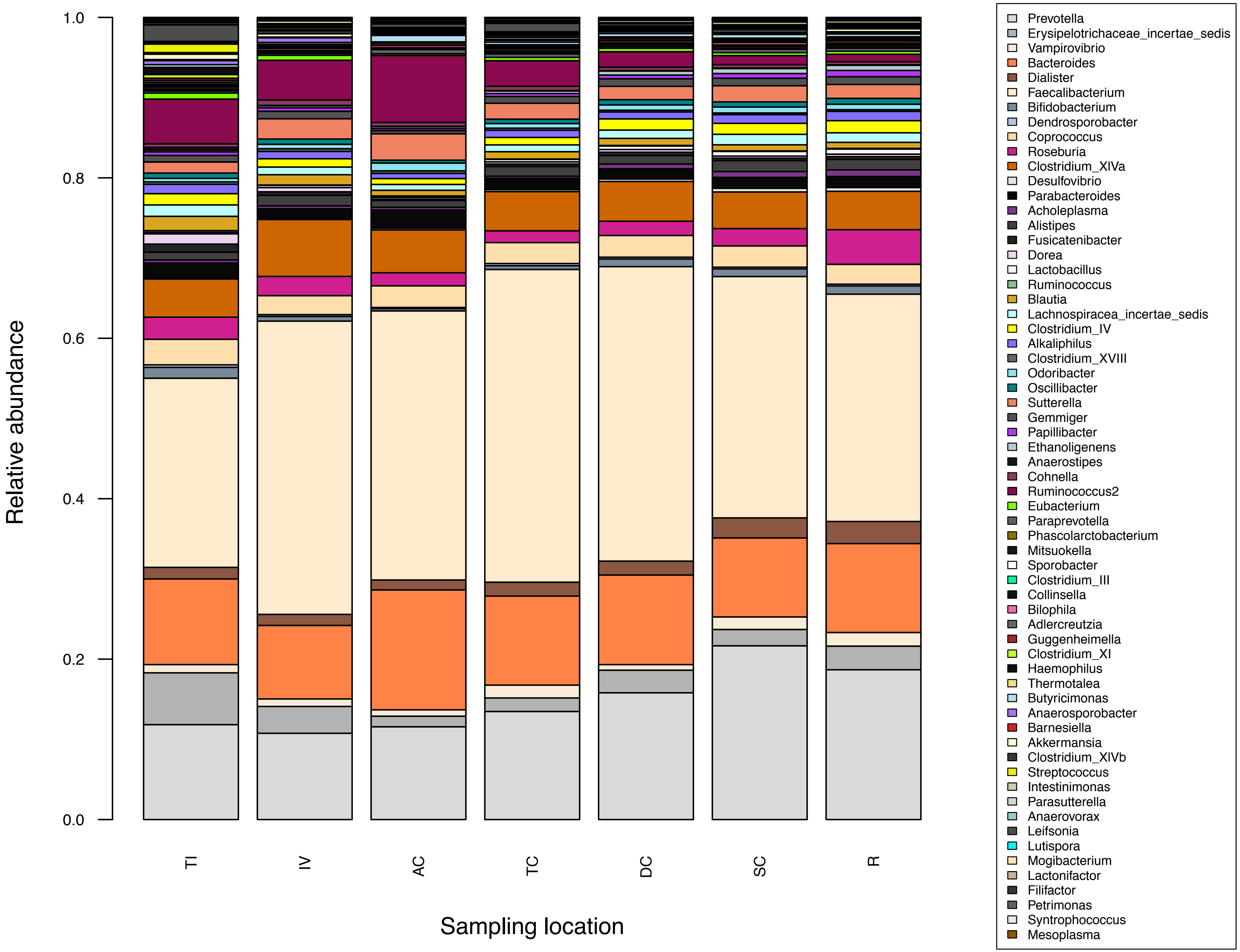
Relative abundance
1.0
0.8
0.6
0.4
0.2
0.0
TI
IV
AC
TC
DC
SC
R
Sampling location
Prevotella
Erysipelotrichaceae_incertae_sedis
Vampirovibrio
Bacteroides
Dialister
Faecalibacterium
Bifidobacterium
Dendrosporobacter
Coprococcus
Roseburia
Clostridium_XIVa
Desulfovibrio
Parabacteroides
Acholeplasma
Alistipes
Fusicatenibacter
Dorea
Lactobacillus
Ruminococcus
Blautia
Lachnospiracea_incertae_sedis
Clostridium_IV
Alkaliphilus
Clostridium_XVIII
Odoribacter
Oscillibacter
Sutterella
Gemmiger
Papillibacter
Ethanoligenens
Anaerostipes
Cohnella
Ruminococcus2
Eubacterium
Paraprevotella
Phascolarctobacterium
Mitsuokella
Sporobacter
Clostridium_III
Collinsella
Bilophila
Adlercreutzia
Guggenheimella
Clostridium_XI
Haemophilus
Thermotalea
Butyricimonas
Anaerosporobacter
Barnesiella
Akkermansia
Clostridium_XIVb
Streptococcus
Intestinimonas
Parasutterella
Anaerovorax
Leifsonia
Lutispora
Mogibacterium
Lactonifactor
Filifactor
Petrimonas
Syntrophococcus
Mesoplasma
